# Age standardisation – an indigenous standard?

**DOI:** 10.1186/1742-7622-4-3

**Published:** 2007-05-14

**Authors:** Bridget Robson, Gordon Purdie, Fiona Cram, Shirley Simmonds

**Affiliations:** 1 Te Rōpū Rangahau a Eru Pōmare (Eru Pōmare Māori Health Research Centre), University of Otago, Wellington, P O Box 7343, Wellington South, New Zealand

## Abstract

The study of inequities in health is a critical component of monitoring government obligations to uphold the rights of Indigenous Peoples. In Aotearoa/New Zealand the indigenous Māori population has a substantially younger age structure than the non-indigenous population making it necessary to account for age differences when comparing population health outcomes. An age-standardised rate is a summary measure of a rate that a population would have if it had a standard age structure. Changing age standards have stimulated interest in the potential impact of population standards on disparities data and consequently on health policy.

This paper compares the age structure of the Māori and non-Māori populations with two standard populations commonly used in New Zealand: Segi's world and WHO world populations. The performance of these standards in Māori and non-Māori mortality data was then measured against the use of the Māori population as a standard. It was found that the choice of population standard affects the magnitude of mortality rates, rate ratios and rate differences, the relative ranking of causes of death, and the relative width of confidence intervals. This in turn will affect the monitoring of trends in health outcomes and health policy decision-making. It is concluded that the choice of age standard has political implications and the development and utilisation of an international indigenous population standard should be considered.

## Background

Epidemiology has a powerful place in an 'evidence-based' policy environment. Trends and disparities in mortality, morbidity and health service receipt or utilisation are used to guide policy, prioritise purchasing, and plan strategies and services in the health sector. In Aotearoa/New Zealand, this information is also used to monitor the impact of government policy and actions on the health of Māori (the indigenous population) and non-Māori (the settler colonial population), and on health disparities between these groups. The imperative to evaluate policy impact on indigenous peoples (see endnote 1), and indeed all ethnic groups, is a function of the right to health[1] and the rights of all indigenous peoples to self-determination and freedom from discrimination in health outcomes[2] (see endnote 2). In Aotearoa/New Zealand the Treaty of Waitangi places specific obligations on the government to ensure that the rights of Māori are upheld.

Accurate monitoring and evaluation of the impact of government policy on Māori and non-Māori health requires high quality data. The evaluation of patient care by ethnicity is an essential element of overall health care quality improvement, resulting in health sector initiatives to improve data collection and utilisation [3-5]. Epidemiology thus has a vital role in the enterprise of ensuring good health outcomes for all peoples. However, the tools used in epidemiology need careful scrutiny to ensure they are as responsive to the interests of indigenous peoples and minority ethnic groups as they are to those of numerically dominant populations.

Kaupapa Māori research is an emerging methodology that centralises indigenous peoples' realities and critiques how Māori are represented in research[6]. The challenge of Kaupapa Māori epidemiology is to operationalise Māori self-determination in health research, to contribute to the right of the people to determine their own future development and priorities, and to respond to the demographic circumstances of the indigenous population.

### Why adjusting for age is necessary

Comparisons of health outcomes between groups or across time periods, where age structures differ, require techniques that adjust for variations in the age structure of populations. This is particularly important for comparing data between Māori and non-Māori populations as the Māori population has a younger age structure. The most informative epidemiological method is to examine differences in age-specific rates but, because the comparison of multiple age-specific rates can be cumbersome, summary measures accounting for differences in age distributions are often used.

An example is given in table 1, using all-cause mortality rates in New Zealand for a five-year time period. The data shows that non-Māori had lower age-specific mortality rates in all age groups but a higher overall crude mortality rate than Māori during 1996–2000. The reason for this is that the non-Māori population has a higher proportion of people in the older age groups where the risk of death is higher and therefore a higher number of deaths in this group. Taken at face value, the crude rates could give the impression that Māori health status was better than that of non-Māori, whereas the age-specific rates actually reveal the reverse of this. If summary measures only were to be used, age-adjusted rates would therefore give a more valid comparison.

**Table 1:** Age-specific all-cause mortality rates in Māori and non-Māori populations 1996–2000

	**Māori**		**Non-Māori**		
		
**Age group**	**Number of deaths**	**Rate per 100,000 per year**	**Number of deaths**	**Rate per 100,000 per year**	**Māori: non-Māori rate ratio**
Under 1 year	777	1047	1033	492	2.13
1–4 years	179	61	266	31	2.00
5–14 years	196	30	387	17	1.73
15–24 years	623	120	1621	75	1.61
25–44 years	1742	221	4922	98	2.26
45–64 years	4543	1324	16871	468	2.83
65 and over	5455	6424	98791	4649	1.38
Total (crude)	13515	490	123891	762	0.64

### Age-standardisation

There are a number of methods that can take account of age differences when comparing health outcomes between different populations, and it is important to understand which tool to use when[7]. This paper focuses on direct age-standardisation, one technique frequently used in New Zealand to monitor disparities in mortality and morbidity rates between the indigenous population and others [8-11]. In this method age-specific rates from *study *population(s) are applied to a *standard *population structure. The expected number of cases is then summed and divided by the total standard population to obtain the overall standardised rate[12]. The weights provided by the standard population are the same in all comparison groups, thus allowing comparison of rates for several study populations and between time periods.

### Effects of different standard populations

The choice of standard population is generally thought to be arbitrary but can nevertheless affect the results obtained. In general the use of a *young *standard leads to a *low *standardised mortality rate, and an *old *standard to a *high *overall rate, due to the strong association between age and mortality[12]. This was demonstrated recently when the United States (US) changed its population standard from one based on the 1940 US population to the significantly older 2000 US population. US coronary heart disease mortality rates for 1994 adjusted using the 1940 age structure gave a rate of 87 per 100,000. However when the rate was calculated two years later in 1996, but standardised to the 2000 age structure, the result was more than twice as high at 187 per 100,000[13]. An effect was also observed in trends over time. Between 1979 and 1995 the crude US death rate increased by 3.3%. Standardised to the 1940 population the rate decreased by 12.6% while that based on the 2000 standard decreased by only 9.2%[13]. Because the decreases in age-specific mortality rates were smaller in the older age groups, the decline appears attenuated in the 2000 standardised rates.

Importantly, racial/ethnic disparities in health were also affected by the change to the 2000 standard, appearing substantially smaller than those standardised to the younger standard[13]. For example, 1995 mortality ratios for African Americans compared to White Americans reduced from 1.6 using the 1940 standard to 1.4 using the 2000 standard. The 2000 standard, driven primarily by the age distribution of the White non-Hispanic population, gives greater weight to older age groups where the age-specific rates for the two populations differ least[14]. Apparent reduced disparities were also observed between the indigenous and general populations in the US, prompting concern that artefactual changes may be mistaken for real progress in eliminating health gaps[15].

In general, choosing a standard population with higher proportions in the younger age groups gives greater weight to events more likely to occur in these age groups such as motor vehicle accidents, infant deaths and youth suicide. Conversely the choice of an older standard gives more prominence to events that are more frequent at older ages, such as deaths from cancer or cardiovascular disease[16]. This could potentially affect perceptions of disparities, prioritising decisions or have other flow-on effects in policy.

### Segi's World and the WHO World Populations

In New Zealand, the most common standard populations used to compare Māori and non-Māori mortality rates have been Segi's world population[17,18] and the recently introduced World Health Organisation (WHO) world population[16].

Segi's "world" population, devised in the late 1950s by cancer epidemiologist Dr Mitsuo Segi, was based on the sum total of male and female populations of the 46 countries in the 1950 publications of the WHO[19]. It was proposed as an intermediate "world" standard, with an age structure between that of the younger "African" standard and the older "European" standard[18]. Segi's "world" and the "European" standards were adopted by WHO in the mid 1960s for calculating age-standardised death rates[16].

The new WHO world standard is based on estimates of the average age structure of the world's population (countries not defined) expected from the year 2000 to 2025. Although younger than the "European" standard, it has fewer children than Segi's standard and a higher proportion aged 70 years and over [16].

The substantial variation in age structure between indigenous and non-indigenous populations of Aotearoa/New Zealand and the recent introduction of the WHO world standard has stimulated interest in the effect of different age standards on Māori health and disparities data. This paper first compares the Māori and non-Māori population age structures with Segi's world population and the WHO world population standards. The two world standards, along with a Māori population standard, are then applied to Māori and non-Māori mortality data and the implications discussed.

## Methods

The Māori population was obtained from Statistics New Zealand's revised estimates of the mid-year resident Māori Ethnic Group population for the years 1996–2000[20]. This was used for denominators in rates and as the Māori standard population. The non-Māori population was constructed by subtracting the Māori population estimates from the total New Zealand population estimates for each year[21]. Deaths registered between 1 January 1996 and 31 December 2000 were obtained from the NZHIS. They were classified as Māori if Māori was coded as one of the ethnic groups in any ethnicity field of the death or cancer registration, hospital admission or the National Health Index (see endnote 3). Otherwise they were classified as non-Māori.

Age-standardised death rates were calculated for Māori and non-Māori. Age-sex-specific rates were calculated by summing the number of deaths in each 5-year age-sex-ethnic group during 1996–2000 and dividing by the sum of the person-years for that age-sex-ethnic group. Each age-sex-specific rate was then multiplied by the standard population weight for that age group and summed to produce the age-sex-standardised rate. Ninety-five percent confidence intervals were calculated for age-sex-standardised mortality rates and ratios using the log-transformation method[22].

## Results

### Comparing Segi's, WHO, Māori and non-Māori populations

Table 2 presents the age distributions of Segi's world population, the WHO world population, and the average Māori and non-Māori populations for 1996–2000. Each cell in Table 2 represents the proportion of the population in that age group, also described as 'weights'. Using the WHO standard, the rate of deaths among 0–4 year olds, for example, contributes only 8.86% to the overall rate, compared to 12.0% in Segi's and 13.29% in the Māori population.

**Table 2:** Percentage of Māori, non-Māori, Segi's world population and WHO world populations in each age group

**Age group**	**Māori Population 1996–2000**	**Non-Māori population 1996–2000**	**Segi's world population**	**WHO world population**
0–4 years	13.29	6.64	12.00	8.86
5–9 years	12.90	7.12	10.00	8.69
10–14 years	11.02	6.75	9.00	8.60
15–19 years	9.94	6.67	9.00	8.47
20–24 years	8.81	6.69	8.00	8.22
25–29 years	8.05	7.26	8.00	7.93
30–34 years	7.60	7.84	6.00	7.61
35–39 years	7.19	8.24	6.00	7.15
40–44 years	5.68	7.58	6.00	6.59
45–49 years	4.47	7.02	6.00	6.04
50–54 years	3.30	6.11	5.00	5.37
55–59 years	2.65	4.96	4.00	4.55
60–64 years	2.03	4.06	4.00	3.72
65–69 years	1.41	3.86	3.00	2.96
70–74 years	0.86	3.47	2.00	2.21
75–79 years	0.46	2.68	1.00	1.52
80–84 years	0.23	1.74	0.50	0.91
85 years and over	0.12	1.31	0.50	0.63*
Total	100.00	100.00	100.00	100.00

* note, the WHO standard has proportions delineated for five year age groups up to the age of 100+, which have been grouped together for the purpose of this comparison.

**Figure 1:**
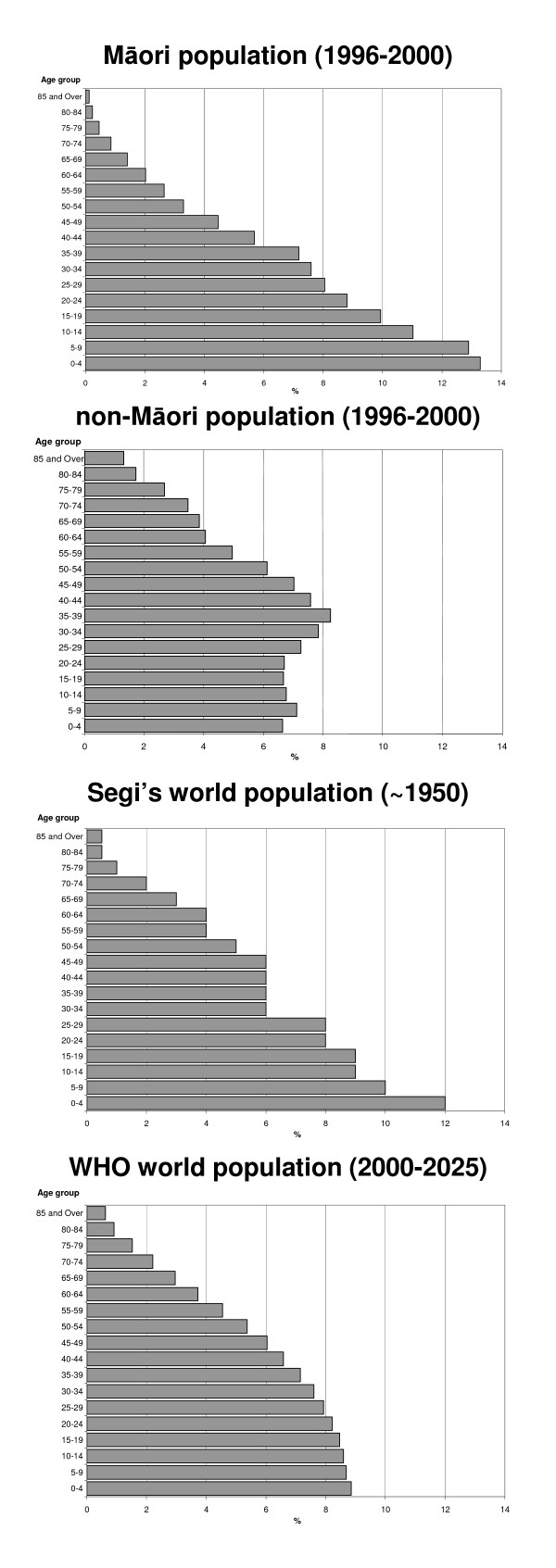
a-d Age structure of the Maori and non-Maori populations of Aotearoa/New Zealand 1996–2000, Segi's world population and WHO world population.

Figure 1 illustrates the differences in overall population structures. The Māori population is younger than any of the other populations, while the non-Māori population is older than any of the other populations. Both Segi's standard and the WHO standard are intermediary between the Māori and non-Māori population age structures. However, Segi's standard is somewhat closer to the Māori population and the WHO standard is closest to the non-Māori structure.

**Figure 2:**
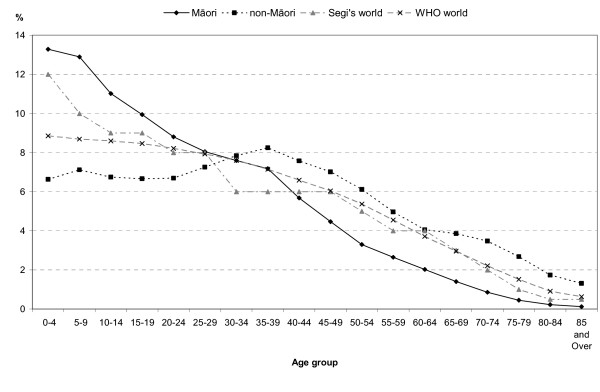
Age-distribution of the Maori and non-Maori average populations 1996–2000, Segi's world population and WHO world population, percent.

Figure 2 illustrates the points of overlap and divergence between the populations. The variation in weights between all four populations is notable in the youngest age groups. The weights are most similar around the 25–44 year age groups.

**Figure 3:**
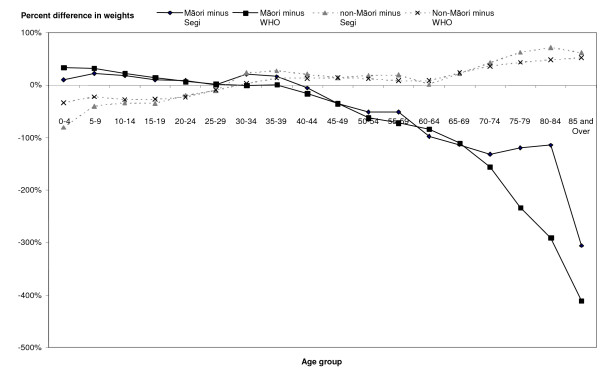
Percent difference in population weights: comparison of Maori and non-Maori populations with Segi's world population and WHO world population.

Large variability in weights between the standard population and the study populations can affect the performance of confidence intervals on rates. The greater the variance, the less adequate the coverage probability of the confidence limits[23]. Figure 3 shows the percentage difference between the age structures of Segi's or the WHO standard populations and the Māori or non-Māori population. In this graph, values closer to zero indicate less difference in weight between the two populations represented. Both Segi's and the WHO standards differ substantially from the Māori population structure in the older age groups. In the very young, Segi's is closer to the Māori weight, while WHO is closer to the non-Māori weight (see table 2). This indicates that the use of either Segi's or the WHO standards will have greater impact on the performance of the confidence intervals for the Māori mortality rates than for the non-Māori rates.

Projections for the Māori and total New Zealand populations to 2021 indicate both populations will age over the next 15 years[24]. The proportion of Māori under 15 years of age is projected to decrease from 37% to 30% and the proportion over 65 years to increase from 3% to 7%. Likewise, the total New Zealand population will show a decrease from 23% to 18% in the young and an increase from 12% to 17% in the senior age group. Nevertheless, the differences between the two populations will remain sizeable.

### The impact of standard populations on Māori and non-Māori mortality data

Table 3 presents crude and age-standardised mortality rates by major cause for Māori and non-Māori during the period 1996–2000. Three sets of adjusted rates are shown: standardised to the average mid-year resident Māori population for 1996–2000, Segi's population and the WHO population. Rates standardised to the Māori population approximate the crude overall rates for Māori. Crude rates for non-Māori are shown in column seven. The table is ranked according to the number of deaths among Māori.

**Table 3:** Age-sex standardised mortality rates by cause, for Māori and non-Māori 1996–2000, standardised to Māori, Segi's and WHO world populations

	**Māori**				**Non-Māori**							
				
		**Rate per 100,000 standardised to:**					**Rate per 100,000 standardised to:**			**Māori to non-Māori rate ratio**		
					
**ICD chapter – ranked by number of Māori deaths**	**No. of deaths**	**Māori pop**	**Segi pop**	**WHO pop**	**No. of deaths**	**Crude rate**	**Māori pop**	**Segi pop**	**WHO pop**	**Māori pop**	**Segi pop**	**WHO pop**
Circulatory system	4552	167	336	402	52074	320	67	152	193	2.49	2.20	2.08
Cancers	3488	128	236	270	34916	215	68	129	151	1.87	1.83	1.79
External causes	1722	63	66	69	6789	42	33	36	38	1.95	1.87	1.83
Respiratory system	1030	38	80	97	12023	74	15	36	46	2.54	2.24	2.13
Endocrine, nutritional & metabolic diseases	1007	37	69	77	3630	22	6	12	15	5.79	5.60	5.26
Symptoms & signs	283	10	10	8	351	2	2	2	2	5.03	4.57	4.24
Digestive system	240	9	17	21	3304	20	5	10	12	1.93	1.77	1.69
Perinatal conditions	219	8	7	5	439	3	5	5	4	1.46	1.46	1.47
Congenital anomalies	200	7	7	6	741	5	6	6	5	1.18	1.19	1.16
Diseases of the nervous system	189	7	10	11	2515	15	6	9	11	1.23	1.04	1.00
Diseases of the genitourinary system	166	6	13	16	1668	10	2	5	6	3.15	2.69	2.52
Infectious & parasitic diseases	155	6	9	10	723	4	2	3	3	2.53	2.93	2.96
Mental & behavioural disorders	144	5	9	11	3239	20	4	8	11	1.48	1.13	1.06
**All-cause**	**13515**	**490**	**877**	**1013**	**123891**	**762**	**219**	**418**	**502**	**2.24**	**2.10**	**2.02**

#### All-cause deaths

The Māori to non-Māori rate ratio for all-cause deaths was highest when derived using the Māori population as the standard at 2.24 (95% CI 2.20–2.28) and lowest using the WHO standard at 2.02 (95% CI 1.98–2.06). Segi's standard produced an intermediate ratio at 2.10 (2.06–2.14) (table 3). The difference in age-standardised rate ratios was due to the greater weight given by the Māori standard to the very young and middle age groups where the age-specific rate ratios are higher (see table 1). The age group with the lowest rate ratio (65 years and over) is given the greatest weight by the WHO standard.

The magnitude of the rates varied according to the standard used, with older age standards producing higher rates. For example, the all-cause mortality rate for Māori was 79% higher using Segi's standard (877 per 100,000; 95% CI 861–893) than the Māori standard (490 per 100,000; 95% CI 492–498) and 107% higher using the WHO standard (1,013 per 100,00; 95% CI 993–1033).

#### Deaths by cause

The effect of the choice of standard population varied by cause of death (table 3). The WHO standard produced higher rates than either Segi's or the Māori populations for causes with a steep age gradient that primarily affect the elderly (circulatory disease, cancers, respiratory, endocrine, digestive, and genitourinary diseases). Rates standardised to Segi's population were generally intermediate between the Māori and WHO population standards for most causes.

#### Ranking of causes of death

The relative ranking of cause of death was also affected by the standard used. When standardised to the Māori population, deaths from external causes ranked third for Māori, but dropped to fifth using the WHO or Segi's standard. 'Symptoms and signs' (mostly sudden infant death syndrome) also ranked higher using the Māori standard at 6^th ^highest for Māori, dropping to 11^th ^place using WHO. The age-distribution of deaths is relatively young for both of these causes, with the younger events receiving higher weighting from the younger population standard. Likewise, perinatal conditions and congenital anomalies ranked higher using the Māori standard.

#### Ratios

Māori to non-Māori rate ratios were highest when rates were standardised to the Māori population for all ICD chapters except for infectious and parasitic diseases and congenital anomalies (table 3). The WHO standard produced the lowest ratios generally, due to the higher weight given to older age groups where the age-specific rate ratios were lower (see table 4). The WHO standard had a larger net effect on the circulatory system than on cancer death rates, as differences in age-specific risks were more extreme for circulatory deaths than for cancer deaths.

**Table 4:** Māori to non-Māori age-specific mortality rate ratios for selected causes, 1996–2000

**ICD Chapter**	**0–14 yrs**	**15–44 yrs**	**45–64 yrs**	**65 yrs and over**
Circulatory system	1.05	2.91	3.74	1.28
Cancer	1.30	1.54	2.04	1.46
External causes	2.89	1.94	1.77	1.03
Respiratory system	3.98	4.19	3.73	1.43
Endocrine, nutritional & metabolic	1.86	2.78	8.33	3.89
Symptoms & signs	6.02	1.62	2.76	1.16
Digestive system	5.20	1.61	2.12	1.16
Perinatal conditions	1.61			
Congenital anomalies	1.29	1.07	1.27	1.15

For deaths from external causes, a high proportion occurred in the 15–44 year age group among both Māori and non-Māori (table 5). This age group receives fairly similar weighting in all standard populations. The net effect of the different age standards was therefore small, although the age-specific rate ratios changed quite dramatically between age groups – from 2.89 in children down to 1.03 in the elderly (table 4).

**Table 5:** Age-specific mortality rates per 100,000 between 1996–2000 for selected causes

**ICD Chapter**		**0–14 years**	**15–44 years**	**45–64 years**	**65 years and over**
Circulatory system	Māori	1.0	30.9	513.7	2797.9
	non-Māori	0.9	10.6	137.3	2180.4
Cancer	Māori	5.5	30.1	453.4	1746.3
	non-Māori	4.2	19.6	221.9	1194.0
External causes	Māori	27.2	88.8	59.4	95.4
	non-Māori	9.4	45.8	33.6	92.7
Respiratory system	Māori	4.8	5.5	79.8	747.8
	non-Māori	1.2	1.3	21.4	523.1
Endocrine, nutritional & metabolic	Māori	1.2	6.0	137.5	524.0
	non-Māori	0.6	2.2	16.5	134.5
Symptoms & signs	Māori	25.8	0.4	1.5	9.4
	non-Māori	4.3	0.2	0.5	8.1
Digestive system	Māori	0.8	1.8	22.4	155.4
	non-Māori	0.1	1.1	10.6	133.6
Perinatal conditions	Māori	21.2			
	non-Māori	13.2			
Congenital anomalies	Māori	15.4	1.8	4.1	5.9
	non-Māori	11.9	1.7	3.2	5.1

Deaths from respiratory disease were relatively uncommon until the middle age group for Māori and the elderly for non-Māori (table 5). Thus the relative risks in the older age groups drive the age-adjusted rates, and the extremely high relative risks of death among Māori at the younger age groups (around 4 times the non-Māori rate) are obscured in age-standardised rates. The use of the Māori standard gives more emphasis to the high disparities in premature deaths from respiratory disease.

#### Rate differences

Rate differences may be used, for example, to assess the contribution of certain causes of death to overall mortality disparities and target interventions for reducing health inequities. The proportion of the overall mortality disparity that could be attributed to specific causes of death (ie. the cause-specific rate difference as a proportion of the overall rate difference) varied relatively little for most major causes of death. For example, circulatory system diseases accounted for 37% of the overall rate difference when the Māori standard was used, compared to 41% using Segi's or the WHO standard. However, there was a substantial variation by age standard in the apparent contribution of external causes to mortality inequalities. When derived from the Māori standard, external causes accounted for almost twice the proportion of the overall disparity (11%) compared to Segi's or WHO standards (around 6%).

#### Confidence intervals

The Māori population standard produced the narrowest confidence intervals on the Māori rates and the WHO standard the widest (table 6). This is due to the fact that the variance on the rate is minimised when the population weights are close to the study population[23]. The converse was observed for non-Māori rates. The confidence intervals on the Māori rate ratios were narrowest when derived from the Māori standard for most causes of death.

**Table 6:** Relative width* of 95% confidence intervals (CI) on age-standardised mortality rates and rate ratios, 1996–2000, by Māori, Segi's and WHO world population standards

	**Width of CI on Māori rates**			**Width of CI on non-Māori rates**			**Width of CI on rate ratios**		
	
	**Māori std**	**Segi std**	**WHO std**	**Māori std**	**Segi std**	**WHO std**	**Māori std**	**Segi std**	**WHO std**
Circulatory system	1.0603	1.0647	1.0675	1.0222	1.0192	1.0183	1.0646	1.0677	1.0702
Cancers	1.0690	1.0737	1.0770	1.0260	1.0229	1.0219	1.0741	1.0774	1.0804
External causes	1.0992	1.1067	1.1122	1.0582	1.0527	1.0508	1.1165	1.1204	1.1245
Respiratory system	1.1314	1.1441	1.1509	1.0479	1.0401	1.0382	1.1412	1.1506	1.1565
Endocrine, nutritional & metabolic	1.1321	1.1381	1.1434	1.0909	1.0748	1.0706	1.1636	1.1596	1.1623
Symptoms & signs	1.2628	1.2864	1.3420	1.3336	1.2874	1.2616	1.4485	1.4287	1.4548
Digestive system	1.2906	1.3142	1.3305	1.0945	1.0790	1.0749	1.3108	1.3279	1.3425
Perinatal conditions	1.3035	1.3035	1.3035	1.2058	1.2058	1.2058	1.3833	1.3833	1.3833
Congenital anomalies	1.3196	1.3323	1.3521	1.1772	1.1675	1.1599	1.3796	1.3854	1.3996
Nervous system	1.3320	1.4189	1.4643	1.1233	1.0933	1.0865	1.3626	1.4348	1.4774
Genitourinary system	1.3600	1.3980	1.4166	1.1349	1.1112	1.1065	1.3945	1.4209	1.4371
Infectious & parasitic diseases	1.3723	1.4326	1.4721	1.2323	1.1830	1.1671	1.4612	1.4872	1.5165
Mental & behavioural disorders	1.3914	1.4729	1.4986	1.1148	1.0809	1.0772	1.4158	1.4843	1.5088

* ratio of upper to lower confidence limits

## Discussion

This study found that the choice of standard population affects the magnitude of age-standardised mortality rates and rate ratios, rate differences, ranking of causes of death, and the variance on the rates. Therefore, the age standard used impacts on disparities data between the indigenous and non-indigenous populations of New Zealand and may subsequently influence health policy.

For many causes of death, the older age standards produced lower rate ratios than the Māori standard. The apparent diminishment of disparities may affect perceptions of their importance. For example, Māori rates of death from mental and behavioural disorders were significantly higher than non-Māori rates using the Māori standard (a rate ratio of 1.48; 95% CI 1.24–1.76) but reduced to a non-significant ratio of 1.06 (95% CI 0.87–1.31) using the WHO standard.

When ranking of conditions or causes of death is used to contribute to decisions on health priorities[25], the outcome will be influenced by the age standard. In this study, deaths from external causes ranked 3^rd ^for Māori using the Māori standard, but 5^th ^using Segi's or WHO. Injury prevention may therefore be given a higher priority with the Māori population standard than with the others. This may also be the case for other conditions that affect younger age groups more than older people, and is likely to affect decisions on the distribution of health resources.

A Kaupapa Māori analysis centralises the experience of the Māori population. Standardising to a Māori population approximates crude overall mortality rates for Māori, more closely representing the real rates (or average risk) for the indigenous population. Rates standardised to the WHO standard, on the other hand, more closely reflect the average mortality rate of the non-Māori population. Similar to other indigenous populations, only 3.5% of the Māori population are aged 65 years or more, compared to 13% of the non-Maori population. The use of the WHO standard thus privileges the colonial population's mortality experience, potentially influencing prioritising decisions and perceptions of disparities between the two populations. The development of an international indigenous population standard could contribute to an epidemiology centred more within the indigenous world. While it may be controversial to introduce another population standard, there is currently no consistent standard used throughout the international literature[13,26,27].

Several issues require further consideration for the development of an indigenous standard population. Firstly, although age-standardised rates are artificial constructs, they reflect underlying patterns of health disparities resulting from differential access to the political, social, economic and environmental determinants of health, and health care resources[28]. It is these factors, rather than age distribution per se, that require attention and intervention. Nevertheless, the monitoring of trends and disparities has a vital role in stimulating public and political determination to address the root and surface causes of health disparities. Krieger and Williams reported an effect of choice of population standard on socioeconomic disparities, with the older age standard having a net effect of reducing the income gradient in mortality [14]. Thus exploration of the impact of population standards on indigenous health data stratified by socioeconomic measures is also necessary.

Secondly, further work needs to be done to examine the effect of age standards on time trends in inequalities – assessing both rate ratios and rate differences. Changes in age-specific rate ratios or rate differences can affect the validity of age-standardised comparisons over time[29]. Trends in age-specific rates should always be examined before drawing conclusions from age-standardised data. In addition, if an indigenous age standard were to be developed the relevance of the current age structure for future decades will need to be considered.

Thirdly, the relationship with age is different for health service utilisation than for mortality. In Aotearoa/New Zealand, measures of health service performance and funding incentives include age-standardised measures such as rates of visits to primary care services[30]. The effect of different age standards on health utilisation data requires further investigation.

Finally, the usefulness of age-standardised data is underpinned by the quality of the data sources. Accurate and complete enumeration of indigenous peoples and of our vital statistics and health events must be of primary concern to users of health data. Associated with the right to self-identification is the right to be counted as Māori in official statistics[31]. This right of course applies to all indigenous peoples[32] and indeed to all ethnic groups[4].

International comparisons of indigenous peoples could aid efforts to improve indigenous health by comparing outcomes of different nations' legal and health care systems, indigenous sovereignty arrangements, social policy contexts, health care quality improvement mechanisms and programmes to eliminate discrimination and institutional racism. Although age-standardisation is only one technique among many used to depict the health of populations, an age standard that more closely reflects the realities of indigenous populations may assist scientists, researchers and policy makers to create more effective decisions leading to reduced or eliminated disparities, a fundamental goal of all policy.

## Endnotes

1. In this article we are using the United Nations definition of indigenous peoples: "Indigenous communities, peoples and nations are those which, having historical continuity with pre-invasion and pre-colonial societies that developed on their territories, consider themselves distinct from other sectors of the societies now prevailing on those territories, or parts of them. They form at present non-dominant sectors of society and are determined to preserve, develop and transmit to future generations their ancestral territories, and their ethnic identity, as the basis of their continued existence as peoples in accordance with their own cultural patterns, social institutions and legal systems."[33]

2. The United Nations Draft Declaration of the Rights of Indigenous Peoples affirms the right of indigenous peoples to self-determination (Article 3), to freedom from adverse discrimination based on indigenous origin or identity (Article 2), and to access to health services without discrimination (Article 24). The right to health is the right of everyone to the enjoyment of the highest attainable standard of physical and mental health. It includes the right to an effective and integrated health system, encompassing health care and the underlying determinants of health (environmental, social, cultural and economic), accessible to all. The study of inequities in health is a critical component of monitoring government obligations in respect of indigenous rights and ensuring Māori are not discriminated against in health outcomes.

3. New Zealand health data are known to have undercounted Māori during the period of analysis [34]. The 'ever Māori' method of classifying Māori used in this study results is a more accurate representation of the number of Māori deaths[27].

## Abbreviations

ICD International Classification of Diseases

NZHIS New Zealand Health Information System

SMR Standardised Mortality Ratio

US United States

WHO World Health Organisation

## Competing interests

The author(s) declare that they have no competing interests.

## Authors' contributions

BR contributed to the study design and drafted the manuscript. GP performed the statistical analysis and contributed to the design of the study. SS helped to draft the manuscript. FC contributed to the study design. All authors read and approved the final manuscript.
